# Prenatal diagnosis and genetic counseling of a paternally inherited microduplication 18q11.1 to 18q11.2 in a chinese family

**DOI:** 10.1186/s13039-022-00617-x

**Published:** 2022-09-01

**Authors:** Juan Chen, Ying Zhang, Mingxi Zhang

**Affiliations:** 1 Reproductive Medicine Center, Renmin Hospital, Hubei University of Medicine, Shiyan, Hubei People’s Republic of China; 2 Prenatal Diagnosis Center, Renmin Hospital, Hubei University of Medicine, Shiyan, Hubei People’s Republic of China; 3 Hubei Clinical Research Center for Reproductive Medicine, Shiyan, Hubei People’s Republic of China; 4 Biomedical Engineering College, Hubei University of Medicine, Shiyan, Hubei People’s Republic of China; 5 Division of Cardiology, Department of Internal Medicine, Wuhan Hospital of Traditional Chinese Medicine, Wuhan, Hubei People’s Republic of China

**Keywords:** Chromosomal microarray analysis (CMA), Microduplication 18q11.1 to 18q11.2, Prenatal diagnosis, Unbalanced chromosome abnormality (UBCA)

## Abstract

**Background:**

Copy number variants are a substantial source of pathogenic or normal genome variations. Chromosomal imbalances of several megabasepair are normally harmful for the affected person. Still, rarely reported are so-called “unbalanced chromosome abnormalities” (UBCAs), which are either losses or gains or equally large genomic regions, but the carrier is only minimally clinically affected even no clinically affected. The knowledge of such UBCAs is imperative also in noninvasive prenatal testing (NIPT) or chromosomal microarray analysis.

**Case presentation:**

A paternally inherited dup(18)(q11.1q11.2) was identified in a over two generations in a Chinese family. The affected region encompasses 25 genes, among which *GATA6* is expressed in fetal endothelial cells and mesodermal cells. *GATA6* duplications and /or mutations have been seen in cases with congenital heart disease but also non-affected individuals, suggesting incomplete penetrance and variable expressivity.

**Conclusions:**

Duplications in the region of chromosome 18q11 have been rare reported previously in clinically healthy persons. Here a further family with an UBCA in 18q11 is added to the literature, suggesting a careful genetic counselling in prenatal diagnosis.

## **Background**

Besides whole chromosome losses or/and gains, microduplications or/and microdeletions are in the focus of prenatal diagnostics [1]. Now noninvasive prenatal testing (NIPT) is generally used in the screening of fetal chromosome aneuploidy [2].

Chromosome karyotype analysis provides an overview of all chromosomes and can identify numerical or/and structural chromosome abnormalities. Chromosomal microarray analysis (CMA) is a method to detect chromosome abnormalities spanning less than 5 Mb by array technology [3].

Besides clearly disease causing chromosomal imbalances there are also rare cases of the unbalanced chromosome abnormalities (UBCAs) [4] and euchromatic variants [5]. Euchromatic variants do not cause clinical symptoms and are often nothing else than cytogenetically visible copy number variants (CNVs), while UBCAs are gains or losses of euchromatic material in the size of megabasepairs, where according to sheer size of the imbalance a severe phenotype would have to be expected. Still, severe phenotypes remain missing in cases characterized as having a UBCA, and carrier of an UBCA show only minor symptoms or no symptoms [5].

Here we report the characterization of a two-generation family with an in GTG-banding cryptic UBCA in 18q11.1 to 18q11.2 of 5.3 Mb. The first hint towards that came from NIPT.

## Case presentation

**Fig. 1:**
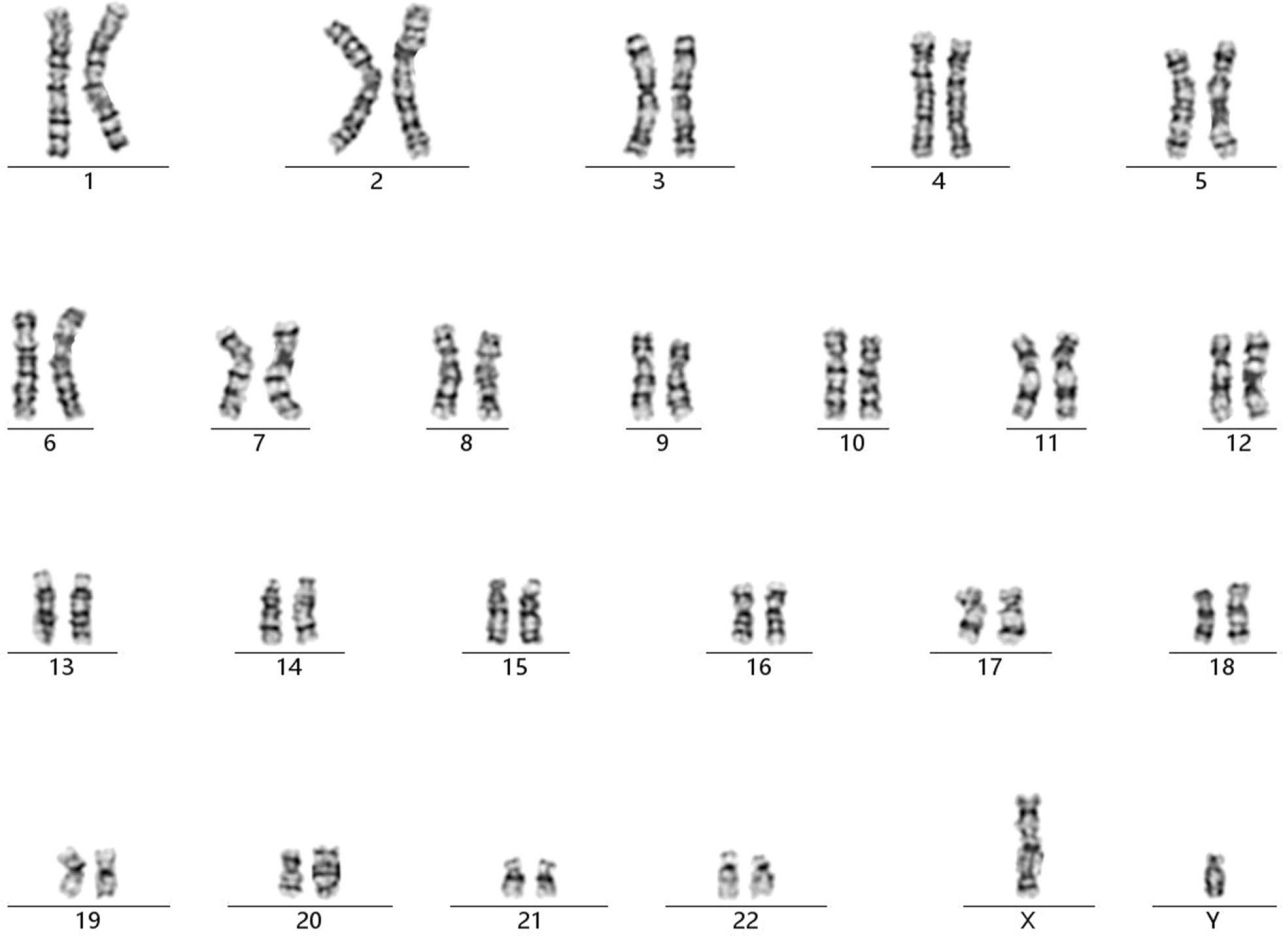
GTG-banding result of the fetus with the cryptic dup(18)(q11.1q11.2)

**Fig. 2:**
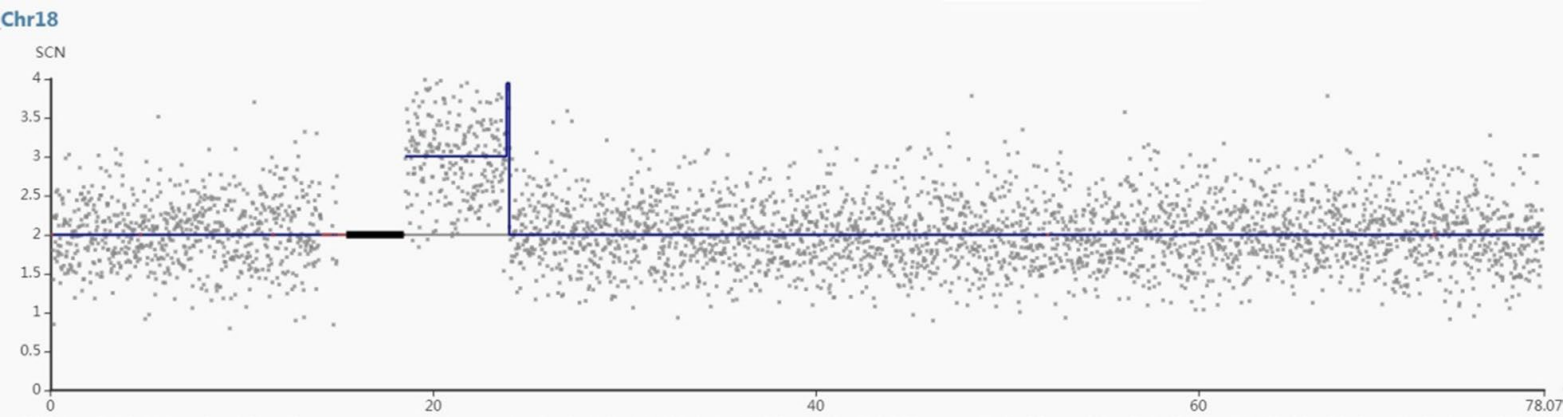
Depiction of the 5.3-Mb duplication revealed by CMA: the final result for the fetus was 46,XY.arr[GRCh37] 18q11.1q11.2(18,540,000_23,840,000)x3

A 41-year-old gravida 1 para 0 female had amniocentesis at pregnancy Week 18 due to the result of a genome wide NIPT screening gave a hint for a 5.3 Mb microduplication encompassing 18q11.1 to 18q11.2. Her husband was 45 years old and no genetic diseases was reported or family history of birth defects. Cytogenetic assessment of amniocyte culture showed a normal male karyotype, 46, XY (Fig. 1). Chromosomal microarray analysis (CMA) assessing uncultured amniocytes was carried out with the Affymetrix CytoScan 750 K chip that comprises 200k SNP markers and 550k non-polymorphic. CMA confirmed the presence of the 5.3-Mb chromosomal duplication, which is to be reported according to International System of Cytogenomic Nomenclature 2020 (ISCN 2020) [6] as arr[GRCh37] 18q11.1q11.2(18,540,000_23,840,000)x3 (Fig. 2).

Both parents had normal karyotypes. However, in CMA the father had the same duplication in 18q as the fetus. Ultrasound examination showed no intrauterine growth restrictions (IUGRs) or congenital malformations except absence of right kidney in the fetus. A comprehensive physical examination of the parents, especially the father showed no abnormalities. The parents decided to continue the pregnancy after genetic counseling. At pregnancy Week 40, a 3500-g boy was born via natural delivery, with Apgar scores of 9/10/10. The baby’s growth parameters at birth were in the normal ranges. The results of complete physical examination were normal except absence of right kidney. At 36-month follow-up, the baby was developing normally (intelligence quotient, IQ = 110).

## Discussion

Duplications about the region of chromosome 18q11 have been rare reported previously in clinically healthy persons. According to the literature [7–10] yet only four cases(in one family) are reported. Here two cases with clearly characterized size of 5.3 Mb is added to the literature. In this family, the fetus and the father have the same duplication, and they both have normal phenotype except absence of right kidney in the fetus. We haven’t been able to confirm whether the absence of right kidney in the fetus is related to the chromosomal duplication. This highlights the necessity to be careful in hasty conclusions about the potential impact of gains or losses as detected in NIPT or CMA analyses. Without a parental genetic test and best also a GTG-banding the nature and impact of a detected imbalance cannot be interpreted reliably.

Still it is interesting and needs further investigations that the in the reported family duplication region in 18q11.1q11.2 encompasses 25 genes as *ROCK1, GREB1L, ABHD3, ANKRD29, AQP4, CABLES1, CABRY, CHST9, CTAGE1, ESCO1, GATA6, IMPACT, KCTD1, LAMA3, MIB1, NPC1, OSBPL1A, PBBP8, RIOK3, RP11, SNRPD1, SS18, TAF4B, TMEM241* and *TTC39C.* In these 25 genes, genetic alterations of ROCK1, ABHD3, ANKRD29, CABLES1 and CTAGE1, including deletion, methylation, and point mutations, are directly involved in the development of human cancer and other diseases [11–13].

*GATA6* is expressed in fetal endothelial cells and mesodermal cells. *GATA6* duplications and /or mutations have been seen in cases with congenital heart disease but also non-affected individuals, suggesting incomplete penetrance and variable expressivity [14].

## Conclusion

With this report it is highlighted that (sub)chromosomal imbalances like microduplication in 18q11.1q11.2 may show great variability concerning phenotypic consequences. UBCA and CNVs identified in prenatal cases need correct interpretations and careful considerations if those are harmful or harmless variants from the norm. Overall, cases like the present remind that parental testing is always necessary, also in cases of imbalances being megabasepairs in size, not to miss UBCAs and terminate a potentially healthy offspring. Our case can be helpful for genetic counseling and prenatal diagnosis. Chromosomal microduplications and microdeletions are difficult to detect by conventional chromosome karyotype analysis. Combination of genetic counseling, chromosome karyotype analysis, CMA and prenatal ultrasound is helpful for the prenatal diagnosis of chromosomal microdeletions/microduplications [15].

## Data Availability

Please contact the corresponding author for data requests.
